# Comparative Efficacy and Toxicity of Modified FOLFIRINOX and Gemcitabine Plus Nab-Paclitaxel in Advanced Pancreatic Ductal Adenocarcinoma: A Real-World Retrospective Analysis

**DOI:** 10.7759/cureus.87389

**Published:** 2025-07-06

**Authors:** Arpit Jain, Varun Goel, Satyajeet Soni, Nivedita Patnaik, Akanksha Jaju, Shaifali Goel, Dharmishtha A Basu, Vineet Talwar, Shivendra Singh

**Affiliations:** 1 Department of Medical Oncology, Rajiv Gandhi Cancer Institute and Research Centre, New Delhi, IND; 2 Medical Oncology, Sri Ram Cancer Centre, Mahatma Gandhi University of Medical Sciences and Technology (MGUMST), Jaipur, IND; 3 Department of Pathology, BLK-Max Super Speciality Hospital, New Delhi, IND; 4 Department of Pathology, ESIC Hospital, Basaidarapur, New Delhi, IND; 5 Department of Surgical Oncology, Rajiv Gandhi Cancer Institute and Research Centre, New Delhi, IND

**Keywords:** chemotherapy, folfirinox, gemcitabine + nab-paclitaxel, indian population, pancreatic cancer

## Abstract

Background

Pancreatic ductal adenocarcinoma (PDAC) remains a highly lethal malignancy with limited effective systemic therapies. Modified FOLFIRINOX (mFOLFIRINOX) and gemcitabine plus nab-paclitaxel (GN) are commonly used first-line regimens. The purpose of this study is to evaluate real-world efficacy (response rates, progression-free survival (PFS) and overall survival (OS)) and toxicity (grade 3/4 hematologic and non-hematologic toxicities) of first-line mFOLFIRINOX and gemcitabine plus nab-paclitaxel in patients with advanced pancreatic ductal adenocarcinoma.

Methods

We conducted a retrospective analysis of 64 patients with advanced PDAC treated between May 2023 and May 2025. Thirty patients received GN, and 34 received mFOLFIRINOX. Efficacy outcomes included overall response rate (ORR), clinical benefit rate (CBR), PFS, and OS. Toxicities were graded using Common Terminology Criteria for Adverse Events (CTCAE) v4.0. Statistical analyses included Kaplan-Meier survival estimates and Cox regression modeling.

Results

The median age was 59 years (range: 32-75), with a predominance of male patients (68.2%). Most had Eastern Cooperative Oncology Group Performance Status (ECOG PS) 0-1 (89.1%). Patients receiving mFOLFIRINOX were younger and more likely to have tumors in the pancreatic head, whereas elevated CA19-9 levels were more common in the GN group.

ORR was 57% in the GN arm and 52.9% with mFOLFIRINOX (P=0.179), while CBR was comparable (77% vs. 76.5%, P=0.985). The median progression-free survival of patients receiving GN was 6.97 months and 8.5 months with FOLFIRINOX (HR: 1.14; 95% CI: 0.58 - 2.22; P=0.713). mFOLFIRINOX had a median overall survival benefit (HR: 1.352; 95% CI 0.63 - 2.90; P=0.428), but this did not reach statistical significance. One-year OS was higher with mFOLFIRINOX (91.6% vs. 82.4%), as was 1.5-year OS (76.3% vs. 55.0%). Paradoxically, one-year PFS favored GN (32.5% vs. 20.3%).

Grade 3/4 hematologic toxicities were more frequent with mFOLFIRINOX (e.g., neutropenia: 20% vs. 8.8%, anemia: 20% vs. 5.9%). GN was associated with more grade 3/4 vomiting (38.2% vs. 10%, P=0.009), diarrhea (26.5% vs. 3.3%, P=0.011), and neuropathy (29.4% vs. 6.7%, P=0.02). Dose modifications and treatment delays were similar, though delays were more frequent in the mFOLFIRINOX arm.

Conclusions

Both mFOLFIRINOX and GN demonstrated comparable efficacy in real-world treatment of advanced PDAC. mFOLFIRINOX offered better long-term OS but carried a higher risk of hematologic toxicity, while GN was associated with greater gastrointestinal and neurological adverse effects. Treatment selection should be guided by patient-specific factors such as comorbidities and tolerance to toxicity. Sequential treatment planning, including access to second-line therapy, significantly impacts survival and should be integral to care strategies.

## Introduction

Pancreatic adenocarcinoma is an aggressive malignancy with limited treatment options, often diagnosed at an advanced stage. Despite improvements in systemic therapy, overall prognosis remains poor. Chemotherapy remains the mainstay of treatment, with modified FOLFIRINOX (mFOLFIRINOX) and gemcitabine plus nab-paclitaxel (GN) being widely used regimens [1]. mFOLFIRINOX has shown survival benefits but with increased toxicity, while GN is considered a more tolerable alternative [2]. This study aims to compare the efficacy and toxicity of mFOLFIRINOX and GN in advanced pancreatic ductal adenocarcinoma (PDAC) patients.

Randomized clinical trials have demonstrated the efficacy of these regimens but little has been done to investigate their effectiveness in the general population, especially in low- and middle-income countries (LMICs) where patient demographics, healthcare systems and supportive care availability differ and may influence outcomes. Furthermore, the differing toxicity burden of each regimen and routine clinical decision making and pragmatic use are pivotal factors in regimen choice. This study will investigate the real-world efficacy (response rates, progression-free survival, and overall survival) and toxicity profiles (grade 3/4 hematologic and non-hematologic toxicities) of mFOLFIRINOX and gemcitabine + nab-paclitaxel as first-line chemotherapy in advanced PDAC, using patient data from a tertiary cancer center.

## Materials and methods

This retrospective analysis was performed at the Rajiv Gandhi Cancer Institute and Research Centre, New Delhi from May 2023 to May 2025 on a total of 64 patients with advanced PDAC. No formal a priori sample size calculation was performed due to the retrospective nature of the study. All eligible patients with histologically proven PDAC with complete treatment and outcome data during this timeframe were included. Patients with incomplete medical records or missing outcome data were excluded.

Eligibility criteria

Inclusion criteria were a histologically confirmed diagnosis of advanced PDAC; receipt of either GN or mFOLFIRINOX as first-line chemotherapy; and availability of complete baseline, treatment, toxicity, and survival data. Exclusion criteria included incomplete medical records or missing outcome data and patients who received both regimens sequentially or concurrently in the first-line setting.

Patients with incomplete clinical records, missing survival or toxicity outcomes, or incomplete treatment documentation were excluded from the final analysis. As a result, the final cohort (n=64) had complete data across all key variables, and no imputation techniques were required.

All clinical, pathological, treatment, and follow-up data were retrospectively extracted from institutional electronic medical records. Radiological response assessments were performed by the treating oncologists in line with institutional imaging protocols, and were assessed using Response Evaluation Criteria in Solid Tumors (RECIST) version 1.1 criteria.

Ethics approval was obtained prior to data analysis and reporting. The Institutional Ethics Committee of Rajiv Gandhi Cancer Institute and Research Centre approved this retrospective study (Approval No.: RES/SCM/66/2024/68; Date: February 18, 2025). Although the study includes patients treated between May 2023 and May 2025, the approval covered retrospective use of all routine clinical data collected within this period. Importantly, no prospective enrollment, intervention, or additional data collection was undertaken after February 2025. Patients treated between February and May 2025 were already undergoing standard-of-care therapy, and their records were included retrospectively. The Institutional Ethics Committee was informed of the full study period.

Baseline information for the patients included age, sex, Eastern Cooperative Oncology Group Performance Status (ECOG PS), disease stage (locally advanced or metastatic), CA19-9 levels, site of the primary tumor, and presence or absence of liver or other metastases. Patients were divided into two groups: 30 patients (47%) received GN; 34 patients (53%) received mFOLFIRINOX. The decision for the patient to receive either regimen resided with the treating oncologist, who would consider the patient's overall health/fitness, comorbidities and anticipated toxicity. Patients who received the sequential or co-administration of both regimens were excluded to preserve internal validity and enable a head-to-head comparison.

The mFOLFIRINOX regimen was delivered biweekly, consisting of oxaliplatin (85 mg/m2), irinotecan (180 mg/m2), leucovorin (400 mg/m2), and 5-fluorouracil (400 mg/m2 bolus and thereafter 2400 mg/m2 continuous infusion over 46 hours). The GN was delivered every 28 days, containing gemcitabine (1000 mg/m2) and nab-paclitaxel (125 mg/m2) delivered on days one, eight, and 15 of each cycle. Dose modifications and treatment delays were at physician discretion and followed by institutional protocols based on hematologic and non-hematologic toxicities.

Radiology imaging for tumor response were assessed every eight to 12 weeks by means of contrast-enhanced CT, with tumor response categories according to RECIST version 1.1 used to evaluate response category as either complete response (CR), partial response (PR), stable disease (SD), or progressive disease (PD). The clinical benefit rate (CBR) was the combination of CR, PR, and SD subjective to receipt of treatment. Adverse events were collected and graded according to the National Cancer Institute Common Terminology Criteria for Adverse Events (NCI-CTCAE), version 4.0.

Progression-free survival (PFS) was defined as the duration of time between the initiation of treatment and either radiographic or clinical progression, or death from any cause. Overall survival (OS) was defined as the time from initiation of treatment to death, or last follow-up.

Statistical analysis

**Figure 1:**
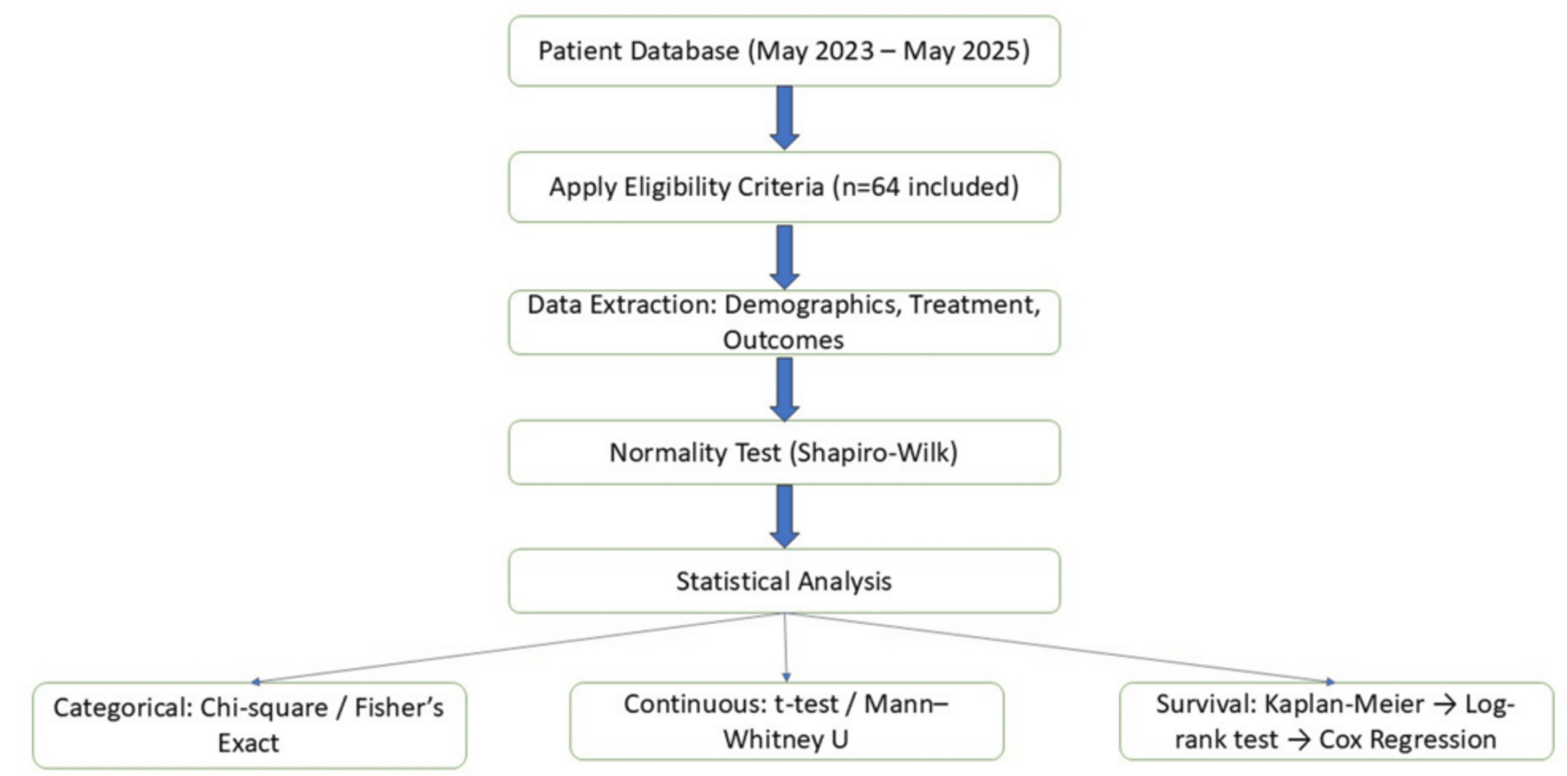
Statistical Analysis Workflow: Flowchart depicting the process of data eligibility screening, statistical method selection, and survival analysis pipeline.

All statistical analyses were conducted using IBM SPSS Statistics for Windows, Version 26.0 (IBM Corp., Armonk, NY, USA). Normality of continuous variables (e.g., age, CA19-9) was tested using the Shapiro-Wilk test. Parametric data were analyzed using the independent t-test, and non-parametric data using the Mann-Whitney U test. Categorical variables (e.g., sex, ECOG PS, tumor site) were compared using the Chi-square test or Fisher’s exact test as appropriate. Survival outcomes (PFS and OS) were estimated using the Kaplan-Meier method and compared using the log-rank test (Figure 1). Patients lost to follow-up or alive at last contact were censored at the date of last follow-up or administrative cut-off date according to standard Kaplan-Meier practices. A two-sided p-value <0.05 was considered statistically significant.

Confounding factor adjustment

To account for potential confounding variables, multivariate Cox proportional hazards regression was performed for both PFS and OS. Covariates included in the model were: age (≥60 vs <60), ECOG performance status, CA19-9 levels, liver metastasis, number of metastatic sites, and treatment regimen. The results of the multivariate analysis are presented in Table 1, which includes hazard ratios (HRs), 95% confidence intervals (CIs), and p-values for each variable.

**Table 1:** Cox Proportional Hazards Regression for Overall Survival (OS) and Progression-Free Survival (PFS) Interpretation: None of the tested covariates reached statistical significance. However, trends toward poorer outcomes were observed for Eastern Cooperative Oncology Group Performance Status (ECOG PS) 2 and higher CA19-9. mFOLFIRINOX: modified FOLFIRINOX; GN: gemcitabine plus nab-paclitaxel

Variable	HR (OS)	95% CI	p-value	HR (PFS)	95% CI	p-value
Regimen (mFOLFIRINOX vs GN)	1.35	0.63–2.90	0.428	1.14	0.58–2.22	0.713
Age (≥60 vs <60)	1.12	0.57–2.19	0.743	1.05	0.52–2.11	0.871
ECOG PS (2 vs 0/1)	2.1	0.85–5.22	0.102	1.95	0.82–4.66	0.138
CA19-9 (>59 vs ≤59 U/mL)	1.78	0.84–3.76	0.132	1.6	0.77–3.35	0.174
Liver Metastasis (Yes vs No)	1.25	0.59–2.64	0.567	1.18	0.56–2.48	0.65
No. of Metastatic Sites (>1 vs 1)	1.62	0.78–3.34	0.181	1.48	0.70–3.15	0.29

## Results

Patient demographics and baseline characteristics

A total of 64 patients with histologically confirmed advanced PDAC were retrospectively evaluated. Of these, 30 patients (46.8%) were treated with GN and 34 patients (53.2%) with mFOLFIRINOX. The cohort had a median age of 59 years (range: 32-75), with a predominance of male patients (n=43, 68.2%). Most had ECOG PS 0-1 (n=57, 89.1%). While there was no significant difference in age distribution (P=0.355), patients in the mFOLFIRINOX group tended to be younger (median 56.5 vs. 60 years). Tumor location showed a significant difference, with 73.5% of patients in the mFOLFIRINOX arm having pancreatic head tumors versus 43% in the GN group (P=0.023). Elevated baseline CA19-9 (>59 U/mL) was significantly more prevalent in the GN group (88.2%, n = 30 vs. 43%, n = 13; P<0.001), possibly indicating a higher tumor burden. Disease status was similarly distributed between groups-78.1%, n = 38 had metastatic disease, while 21.9%, n = 14 had locally advanced disease. Liver metastasis was present in 56.2%, n = 36 of patients, with no significant intergroup difference. Baseline demographic and clinical characteristics of the two treatment groups are summarized in Table 2.

**Table 2:** Baseline Demographic and Clinical Characteristics of the Two Treatment Groups Data are presented as N (%), Mean ± SD, and Median (Range). P-values <0.05 were considered statistically significant. Statistical tests applied include Chi-square test, Fisher’s exact test, and independent t-tests, as appropriate based on distribution and cell counts. ECOG PS: Eastern Cooperative Oncology Group Performance Status; mFOLFIRINOX: modified FOLFIRINOX

Patient Characteristics	Variables	ALL, n (%)	Gemcitabine Nab Paclitaxel, n (%)	mFOLFIRINOX, n (%)	Sig.	Exp(B)	95% C.I. for EXP(B)	P Value
Age (In years)	Mean (SD)	56.6 (9.2)	58.17 (10.01)	55.4 (8.34)	0.761	1.001	0.993, 1.010	
Median (Range)	59 (32-75)	60 (34-75)	56.5 (32-70)					
<60	38 (60.3)	14 (46.7)	20 (58.83)					
>=60	25 (39.6)	16 (53.33)	14 (41.17)		0.695	0.857	0.396, 1.853	
Sex	Male	43 (68.2)	18 (60)	25 (73.53)				0.250
	Female	20 (31.7)	12 (40)	9 (26.47)	0.514	0.750	0.316, 1.780	
ECOG PS at presentation	ECOG PS 0/1	57 (89.1)	26 (87)	31 (91.18)	0.706	0.750	0.168, 3.351	
	ECOG PS 2	7 (10.9)	4 (13)	3 (8.82)				
CA19-9 levels (U/mL)	Normal	6 (9.4)	6 (20)	00 (0.00)				< .001>
	<59	15 (23.4)	11 (37)	4 (11.76)	0.083	0.364	0.116, 1.142	
	>59	43 (67.2)	13 (43)	30 (88.24)	0.012	2.308	1.204, 4.424	
Disease Status	LA	14 (21.87)	6 (42.8)	8 (57.1)				0.733
	Metastatic	38 (78.12)	24 (63.1)	26 (68.4)	0.109	0.583	0.302, 1.128	
Liver Metastasis	No	28 (43.75)	13 (46.4)	15 (53.5)				0.949
	Yes	36 (56.25)	17 (47.2)	19 (52.7)	0.123	0.529	0.236, 1.188	
No. of Metastatic Sites	1	12 (18.75)	6 (50)	6 (50)	0.020	0.333	0.132, 0.840	0.20
	2	44 (68.75)	18 (40.9)	26 (59)	1.000	1.000	0.323, 3.101	
	>2	8 (12.5)	6 (75)	2 (25)	0.999	1.615	0.657, 2.850	
Primary Site	Head	38 (59.4)	13 (43)	25 (73.53)				
	Body	19 (29.7)	14 (47)	5 (14.71)	0.048	0.357	0.129, 0.992	0.023
	Tail	6 (9.4)	2 (7)	4 (11.76)	0.423	2.000	0.366, 10.919	
	Diffuse	1 (1.6)	1 (3)	00 (0.00)	1.000	0.000	0.000	

Treatment response and disease control

The overall response rates (ORR = CR + PR) were comparable between groups (57% in the GN arm and 64.6% in the mFOLFIRINOX arm, p = 0.56). Complete response was seen in 11.7% of patients on the mFOLFIRINOX and no patients on GN (p = 0.112). The disease control rates (DCR = CR + PR + SD) were indistinguishable between groups (77% in GN and 76.5% in mFOLFIRINOX, p = 0.96), which shows that the tumor control was very similar despite the dissimilar toxicity profiles. Best response and disease control of the two treatment groups are summarized in Table 3.

**Table 3:** Best Response and Disease Control. DCR (CR + PR + SD). Comparison of tumor response outcomes between patients treated with gemcitabine + nab-paclitaxel (GN) and modified FOLFIRINOX (mFOLFIRINOX) as first-line chemotherapy for advanced pancreatic ductal adenocarcinoma (PDAC). CR: Complete Response, PR: Partial Response, SD: Stable Disease, PD: Progressive Disease, DCR: Disease Control Rate (CR + PR + SD). No statistically significant differences in complete or partial response rates were observed between groups. The disease control rate was comparable between the GN (77%) and mFOLFIRINOX (76.5%) arms (p = 0.96). p-values were calculated using Chi-square or Fisher’s exact test due to small cell counts, particularly where zero responses were observed in one group.

Response Category	GN Group (n=30)	mFOLFIRINOX Group (n=34)	P-value
Complete Response (CR)	0%	11.7%	0.112
Partial Response (PR)	57%	52.9%	0.770
Stable Disease (SD)	20%	11.8%	0.400
Progressive Disease (PD)	23%	23.5%	0.960
Overall Disease Control	77%	76.5%	0.960

Survival analysis

Landmark analysis showed divergence in OS between the regimens. At 12 months, OS was 91.6% (n=31) in the mFOLFIRINOX group versus 82.4% (n=25) in the GN group. By 18 months, OS rates declined to 76.3% (n=26) and 55.0% (n=17), respectively, favoring mFOLFIRINOX for long-term disease control; however, it remained statistically not significant (HR = 1.35; 95% CI: 0.63-2.90; p = 0.428). Interestingly, one-year PFS was higher with GN (32.5%, n=10) compared to mFOLFIRINOX (20.3%, n=7), suggesting better early disease control with GN. Landmark survival outcomes including PFS and OS at multiple time points are summarized in Table 4.

**Table 4:** Landmark Survival Outcomes Data presented as median (95% CI) or %. P-values <0.05 were considered statistically significant. Log-rank test used for survival comparisons.

Parameter	Gemcitabine + Nab-Paclitaxel	Modified FOLFIRINOX
Median Progression-Free Survival (PFS)	6.97 months (95% CI: 4.60–9.35)	8.50 months (95% CI: 7.04–9.96)
1-Year Overall Survival (OS)	82.4%	91.6%
1.5-Year Overall Survival (OS)	55.0%	76.3%
1-Year Progression-Free Survival	32.5%	20.3%

The median PFS was 6.97 months (95% CI: 4.60-9.35) in the GN group and 8.5 months (95% CI: 7.04-9.96) in the mFOLFIRINOX group. For the overall cohort, median PFS was eight months (95% CI: 6.23-9.77). Despite the numerical advantage in mFOLFIRINOX, the difference was not statistically significant (HR: 1.14; 95% CI: 0.58 - 2.22; P=0.713).

**Figure 2:**
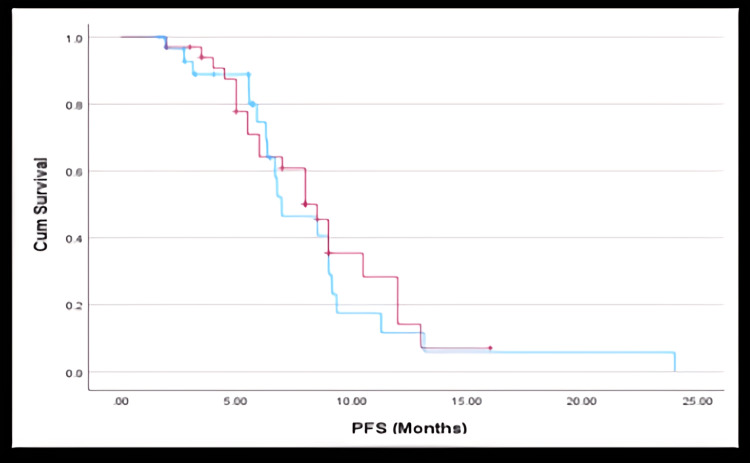
Progression-Free Survival (PFS) Kaplan-Meier curve showing PFS for patients treated with gemcitabine + nab-paclitaxel (GN) and modified FOLFIRINOX (mFOLFIRINOX). Events: 21 in GN group, 24 in mFOLFIRINOX group. Censored observations are marked. No statistically significant difference was observed between the groups (HR: 1.14; 95% CI: 0.58 - 2.22; P=0.713). GN cohort is represented in blue, and mFOLFIRINOX cohort is represented in red.

**Figure 3:**
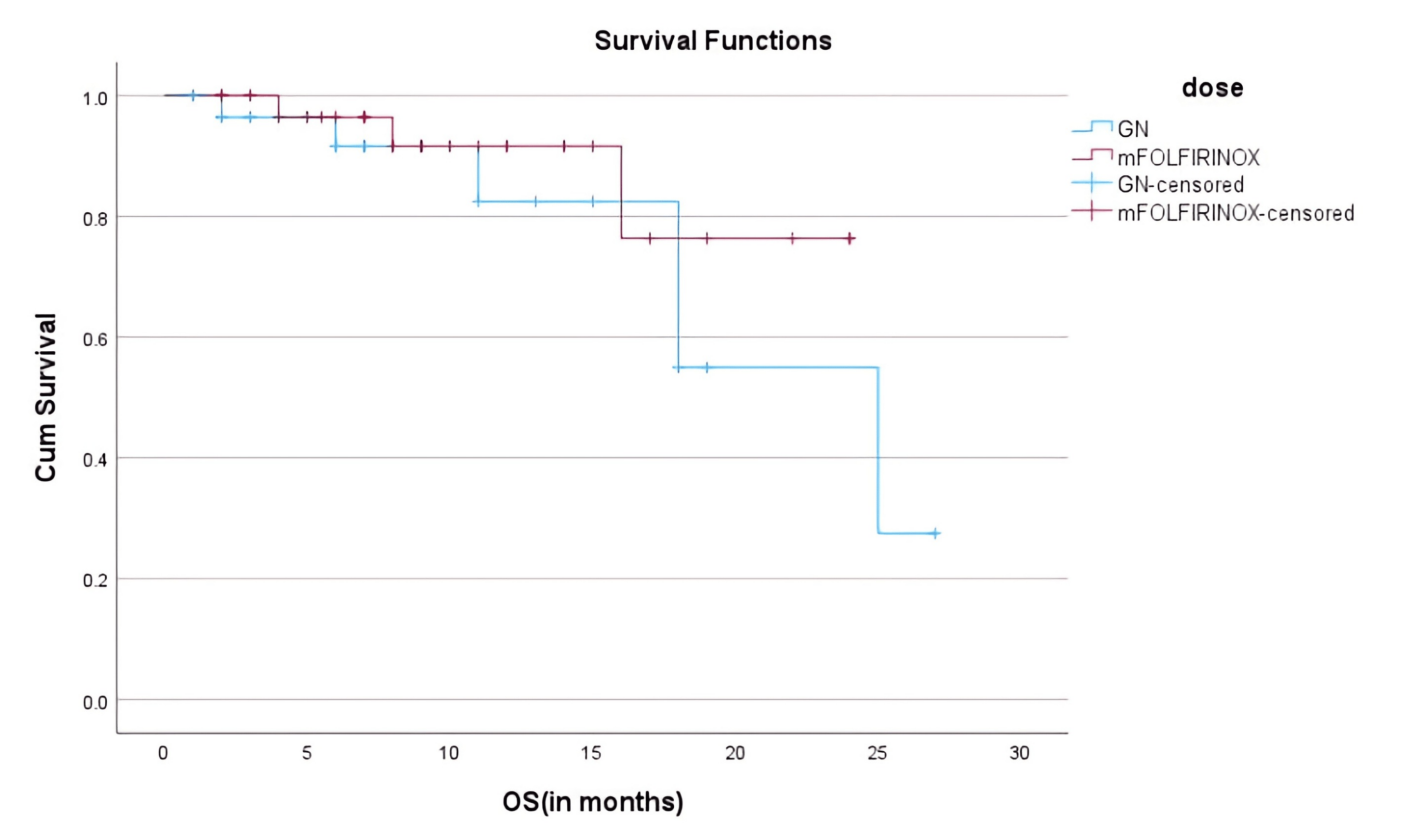
Overall Survival (OS) Kaplan-Meier curve for overall survival in GN (n = 30) vs mFOLFIRINOX (n = 34). Deaths: 15 in GN group, 11 in mFOLFIRINOX group. 1.5-year OS: 55% vs 76.3%. HR = 1.35; 95% CI: 0.63–2.90; p = 0.428.Censored data points are indicated. The mFOLFIRINOX group showed improved long-term survival, though the difference was not statistically significant. mFOLFIRINOX: modified FOLFIRINOX; GN: gemcitabine plus nab-paclitaxel GN cohort is represented in blue, and mFOLFIRINOX cohort is represented in red.

Kaplan-Meier survival plots showed a parallel descent for both groups, with overlapping confidence intervals, highlighting the comparable disease control efficacy of both regimens. As shown in Figure 2, Kaplan-Meier analysis of PFS revealed overlapping curves for GN and mFOLFIRINOX groups, with no significant difference. Similarly, Figure 3 demonstrates a trend toward improved OS in the mFOLFIRINOX group, though this was not statistically significant.

Toxicity and dose modifications

The toxicity profile varied distinctly between regimens. mFOLFIRINOX was associated with more hematologic toxicities, particularly neutropenia (20%, n=7 vs. 8.8%, n=3; P=0.199) and anemia (20%, n=7 vs. 5.9%, n=2; P=0.133). Leukopenia and thrombocytopenia were marginally more frequent in mFOLFIRINOX but not significant. Conversely, GN had a higher incidence of non-hematologic adverse events, including grade 3/4 vomiting (38.2%, n=13 vs. 10%, n=3; P=0.009), diarrhea (26.5%, n=9 vs. 3.3%, n=1; P=0.011), and peripheral neuropathy (29.4%, n=10 vs. 6.7%, n=2; P=0.02). Incidence of common adverse events in the two treatment groups are summarized in Table 5.

**Table 5:** Incidence of Common Adverse Events Frequency of grade 3/4 adverse events in patients receiving GN (n = 30) vs. mFOLFIRINOX (n = 34) and associated odds ratios from logistic regression. Exp(B) values represent the odds of experiencing the adverse event in the GN group compared to mFOLFIRINOX. p-values were calculated using Fisher’s exact test. Statistically significant values (p < 0.05) are bolded. mFOLFIRINOX: modified FOLFIRINOX; GN: gemcitabine plus nab-paclitaxel

Adverse Event (Grade 3/4)	mFOLFIRINOX (n = 34)	GN (n = 30)	p-value	Exp(B)(GN vs. mFOLFIRINOX)	p (logistic)
Neutropenia	7 (20.6%)	3 (8.8%)	0.199	2.4	0.04
Anemia	7 (20.6%)	2 (5.9%)	0.133	–	–
Leukopenia	11 (32%)	9 (30%)	NS	–	–
Thrombocytopenia	10 (29.4%)	8 (26.7%)	NS	–	–
Vomiting	13 (38.2%)	3 (10.0%)	0.009	5.8	0.01
Diarrhea	9 (26.5%)	1 (3.3%)	0.011	6.4	0.02
Peripheral Neuropathy	10 (29.4%)	2 (6.7%)	0.02	–	–

The trends observed in patient-reported adverse events were confirmed through logistic regression modeling. The GN arm was more likely to have a grade 3/4 vomiting (Exp(B) = 5.8, p = 0.01) and diarrhea (Exp(B) = 6.4, p = 0.02) events, and mFOLFIRINOX was significantly more likely to have increased odds of neutropenia (Exp(B) = 2.4, p = 0.04). Peripheral neuropathy, though not modeled in regression, was clearly more prevalent in the mFOLFIRINOX cohort and may reflect cumulative toxicity from multi-agent chemotherapy.

Dose reductions were required for 23% of patients in the GN arm (n=7) and 23.5% of patients in the mFOLFIRINOX arm (n=8)(p=0.985). However, the mFOLFIRINOX arm had increased frequency of treatment delays (76.5%, n=26 vs. 53%, n=16); reason for treatment delays was predominantly hematologic suppression in the mFOLFIRINOX group. Patients in the GN group also experienced delays in treatment predominantly due to gastrointestinal toxicities. Table 5 contains a detailed summary of adverse events by grade.

Second-line therapy and its impact on survival

Of the total cohort, 41 patients proceeded to second-line therapy post-progression. Those receiving further treatment had a significantly better median OS for those who did not. This survival advantage was consistent across both initial treatment arms. The decision to initiate second-line therapy was influenced by initial response, ECOG status, and tolerability. Patients with favorable early responses were more likely to tolerate and access subsequent lines of treatment, underscoring the importance of personalized treatment planning and longitudinal care.

Integrated interpretation

Both GN and mFOLFIRINOX emerged as effective first-line options for advanced PDAC, delivering comparable disease control in the real-world setting. While mFOLFIRINOX showed superior long-term OS, it came at the cost of greater hematologic toxicity. GN, offering better one-year PFS and tolerability in early phases, was associated with more gastrointestinal and neuropathic adverse effects. This study highlights the critical role of individualizing treatment decisions based on patient characteristics: age, ECOG PS, organ function, and anticipated toxicity profile. Moreover, the significant survival benefit from second-line therapy underscores the necessity for continuous treatment planning and early intervention.

## Discussion

This retrospective study provides real-world evidence on the comparative efficacy and toxicity of mFOLFIRINOX and GN as first-line treatments for advanced PDAC. While randomized controlled trials have established these regimens as standard options [1,2], direct comparative data in routine clinical practice remain limited, particularly from the Indian subcontinent. Our findings add valuable insights into regimen selection, survival trends, and tolerability in a non-trial population.

The PRODIGE-4/ACCORD trial demonstrated the superiority of FOLFIRINOX over gemcitabine monotherapy in metastatic PDAC, reporting a median OS of 11.1 months versus 6.8 months, respectively [1]. In contrast, the MPACT trial showed that GN offered a median OS of 8.5 months, compared to 6.7 months with gemcitabine alone [2]. These trials led to widespread adoption of mFOLFIRINOX and GN as first-line options, with regimen selection often based on patient fitness and toxicity profiles [3].

Our real-world study observed a median PFS in our study was 6.97 months (GN) vs. 8.5 months (mFOLFIRINOX). These findings are consistent with retrospective analyses from other regions [4-6]. For instance, a Canadian real-world study by Gourgou-Bourgade et al. reported similar PFS durations of 7.2-9 months for mFOLFIRINOX and six to seven months for GN [4]. Similarly, Korean and European cohorts have found comparable outcomes in non-trial populations [5-7].

In our cohort, the ORR for GN was slightly higher than mFOLFIRINOX (57% vs. 52.9%), although not statistically significant. The CBR was almost identical across both groups. These results mirror a study by Portal et al. from France, which reported CBRs of approximately 75% for both regimens in a non-trial setting [7].

Interestingly, in subgroup analysis, we observed better disease control among patients with pancreatic head tumors, particularly in the mFOLFIRINOX group. Though speculative, this may be related to tumor biology or anatomical differences affecting drug delivery [8]. This observation warrants further investigation in prospective trials.

Toxicity profiles differed significantly between the two regimens in our study. As in the PRODIGE-4 trial, mFOLFIRINOX was associated with higher rates of hematologic toxicities, notably grade 3/4 neutropenia and anemia [1]. GN, on the other hand, led to more non-hematologic toxicities, such as gastrointestinal side effects and neuropathy, consistent with findings from the MPACT trial and other global studies [2,7,9]. Despite these differences, dose modifications and treatment delays were similarly frequent between groups (GN: 23%, mFOLFIRINOX: 23.5%), suggesting that with proper management and dose tailoring, both regimens can be safely administered in real-world settings [10].

The two arms were similarly effective overall with mFOLFIRINOX potentially offering longer-term OS. Differences in toxicity between arms and a patient's performance status and existing comorbidities often guide treatment selection. GN was tolerated better by older or fragile patients.

The toxicity profiles reported here are consistent with what is known about the pharmacologic properties of both regimens and inferences to support clinical decision making. mFOLFIRINOX was associated with significantly higher odds of both grade 3/4 neutropenia (Exp(B) = 2.4, p = 0.04), vomiting (Exp(B) = 5.8, p = 0.01) and diarrhea (Exp(B) = 6.4, p = 0.02) elevating the hematologic and gastrointestinal burden of risk. Although outside of regression modeling, peripheral neuropathy was also significantly more frequent in the mFOLFIRINOX group and likely related to cumulative doses of oxaliplatin. These toxicities directly impacted the way the treatment was modified, with hematologic toxicities (primarily neutropenia and anemia) being the most common cause of dose delay in mFOLFIRINOX and gastrointestinal side effects (vomiting and diarrhea) as the most common reason for treatment interruptions in GN. Appreciating the differential toxicity findings, the regimens should be selected not only because of the characteristics of the disease but also by what individual patients are able to tolerate and how best to support them, especially when resources are limited.

A notable contribution of our study is the landmark survival analysis, revealing that although GN offered better early PFS at one year (32.5% vs. 20.3%), mFOLFIRINOX was superior in terms of long-term OS, with 76.3% of patients alive at 1.5 years compared to 55.0% in the GN group. This trend suggests that while GN may be more effective in early disease control, mFOLFIRINOX offers more durable survival benefits, likely due to deeper responses and longer PFS in select patients [11-13].

These results align with findings from the NAPOLI-1 and FIREGEM studies, which emphasized the importance of sustaining disease control and sequencing therapy in advanced PDAC [11,12]. Moreover, real-world datasets such as those from Carrato et al. and Tempero et al. underscore the economic and quality-of-life implications of treatment decisions [10,6].

Real-world implications and clinical applicability

The real-world applicability of these findings is high. Unlike trial populations, which are often highly selected, our study included patients with a broader range of age, performance status, and comorbidities. Thus, our results better reflect routine oncology practice, especially in developing countries [3,14,15].

We recognize that baseline imbalances may have contributed to treatment outcomes. Patients assigned to the GN group had higher CA19-9 levels and somewhat worse performance status at baseline, both of which may have influenced both the selection of the initial regimen chosen for that cohort and their overall prognosis. Also, limited availability or eligibility for second-line therapy in this group may further influence survival outcomes. These observations echo the reality of modern treatment; in patients with more severely advanced disease or who are more frail, regimens are often less intensive, and optimal sequential therapy may not be undertaken. Ultimately, these experiences highlight the importance of patient fitness, disease burden, and health systems’ ability to deliver a real-world survival experience.

This study emphasizes the need for patient-centric regimen selection. For younger, fit patients with high disease burden, mFOLFIRINOX may provide superior survival at the cost of increased toxicity. Conversely, GN is a preferred option for patients with borderline performance status or comorbidities, given its tolerability and early disease stabilization potential [14,8].

While overall survival differences between the two treatments were not statistically significant, the mFOLFIRINOX arm showed a tendency towards long-term benefit. Patient selection bias may play a role, as younger, healthier patients with better performance status and fewer comorbidities were more likely to be offered mFOLFIRINOX. Additionally, biologic differences between groups such as less disease burden, location of the tumor (head versus body/tail of pancreas), or differences in genomic alterations to allow differences in survival. The potential confounding nature of these factors and the retrospective nature of the study reemphasize the limitations of retrospective design and the need to be cautious in the interpretation of outcome trends.

In the future, the use of molecular and genomic biomarkers may be helpful to further refine treatment selection in advanced PDAC. For example, BRCA1/2 mutations and homologous recombination deficiency (HRD) are being explored as predictive markers for platinum sensitivity and PARP inhibitors for maintenance. Furthermore, beginning maintenance strategies early (for example, using fluoropyrimidine-based therapy in the maintenance phase following the initial de-escalating induction) may help to optimize treatment efficacy with acceptable long-term tolerability. Although potentially limited by access and affordability in the LMIC context, these evolving approaches represent important future directions for individualized therapy.

Limitations

Our study has several limitations. First, its retrospective nature introduces the risk of selection and information biases. Second, the sample size was relatively small, limiting the statistical power to detect differences in some outcomes. Third, it was conducted at a single tertiary center, which may affect generalizability. Another limitation was the lack of BRCA mutation status or other molecular markers that may have influenced treatment decision-making and survival outcomes, but were not routinely evaluated in our cohort. Furthermore, alopecia was excluded from the adverse event profile, as it was not consistently documented in the electronic medical record at the time of treatment.

Future directions

Future prospective studies should aim to validate our findings in larger, multicentric cohorts. Importantly, biomarker-guided selection of first-line therapy, such as HRD testing, CA19-9 kinetics, or transcriptomic subtyping, should be incorporated into real-world algorithms to optimize outcomes. In addition, real-time adaptation of therapy based on response (e.g., switch maintenance strategies) may further enhance survival in this challenging malignancy.

## Conclusions

This real-world study indicates that modified FOLFIRINOX and gemcitabine plus nab-paclitaxel are both valid first-line treatment options and each treatment has its distinct advantages. It is clear that there are advantages and disadvantages to mFOLFIRINOX which had significantly improved long-term overall survival, but more hematologic toxicity. GN showed improved early disease control with better tolerability in some patients with marginal performance status.

The choice in chemotherapy should depend on the individual patient factors such as performance status, comorbid disease, tumor burden, and expected tolerability. Equally important, this study highlighted the importance of access to second-line therapy when thinking about overall improvement in patient outcomes, and the necessary longitudinal, continued and active treatment of PDAC. Future prospective studies incorporating biomarker-based stratification of patients and allowing real-time changes in therapy need to be performed to improve treatment strategies and survival in this difficult-to-treat cancer.
